# Survival of histologically proven carcinoma of the lung registered in the North West Thames Region, 1975-1979.

**DOI:** 10.1038/bjc.1982.316

**Published:** 1982-12

**Authors:** R. F. Mould, R. J. Williams


*Br J. Cancer* (1982) **46**, 999

### *Short Communication*

### SURVIVAL OF HISTOLOGICALLY PROVEN CARCINOMA OF THE LUNG REGISTERED IN THE NORTH WEST THAMES REGION, 1975–1979

R. F. MOULD AND R. J. WILLIAMS

*From the Department of Medical Physics, Westminster Hospital, Page Street Wing, London SW1P 2AP*

Received 5 August 1982 Accepted 7 October 1982

LUNG CANCER survival statistics published by the Office of Population Censuses and Surveys (OPCS, 1980, 1982) for 1971–1975 for England and Wales have not been classified into untreated and treated groups or into histological groups. In addition, unlike the lung cancer survival figures prepared by the End Results Section of the Biometry Branch of the National Cancer Institute, now part of the Cancer Surveillance, Epidemiology and End Results (SEER) program of the National Cancer Institute (USDHHS, 1976), there is currently no attempt by OPCS to distinguish between those registrations with and without histological confirmation. This is a disadvantage in that registrations without any histological confirmation cannot be *positively* identified as true cancer cases. This study of 3706 histologically confirmed cancer registrations in the North West Thames Region thus provides survival data for sub-groups not at present analysed by OPCS although the raw data are available on an OPCS cancer abstract card. Excellent follow-up was achieved in that 97·5% of all cases were successfully followed-up in 1981–1982.

The North-West Thames Region had an estimated home population as at 30 June 1977 of $1{\cdot}6593 \times 10^6$ males and $1{\cdot}7731 \times 10^6$ females and represents 7% of the total population of England and Wales. It is the fourth most highly populated of the 15 regions after the West Midlands (10·5%), Trent (9·2%) and the North Western (8·2%) and cancer registrations are submitted from some 40 hospitals to the regional registry.

Data were abstracted by one of us (R.J.W.) from the OPCS cancer abstract cards held in the registry. On this card information is required for *Treated at this hospital* and for *Histology confirmed.* These data were first explicitly requested on the 1976 OPCS cancer abstract card. Prior to 1976 the information *Type of growth* was requested only in the form *Clinical type of growth* and *Histological type of growth, if known.* Histological confirmation of squamous cell carcinoma, oat cell carcinoma and adenocarcinoma could therefore be assessed for the 1975 records but whether or not a 1975 registered patient received treatment could only be assessed for 3 hospitals in the region. Two, Harefield and Mount Vernon, recorded this information for 1975 in the space on the abstract card headed *Notes* and for Westminster the information was recorded on the reverse of the abstract card copy retained by Westminster. Data abstraction was for males and females for 1975–1977 and additionally for females 1978–1979. The sex ratio males : females for lung cancer in England and Wales in 1977 (OPCS, 1982) is 3·7 : 1 and the additional 2 years of records for females were obtained to increase the sample size for analysis, otherwise a sub-grouping by histology for females would not have been possible. For the 2 years 1976–1977 with full data for all hospitals and both sexes the sex ratio in the region for histologically confirmed registrations was 3·5 : 1.

It would obviously have been impossible in a short space of time to write for follow-up to, and receive a reply from, the appropriate hospital record departments and general practitioners for those of the 3706 registrations who, according to the North-West Thames Regional Cancer Registry records, had not died. However, by tracing each case using the NHS number and the facilities of the National Health Service Central Registry at Southport, follow-up on whether dead or alive could be rapidly obtained. Hence, following record abstraction from the abstract cards in the regional registry, an initial follow-up was obtained from the NHS Central Registry in October 1980 with a final follow-up in February 1982. In addition, all those registrations alive subsequent to NHS Central Registry follow-up were also subjected to a follow-up enquiry to the cancer registration officers at the hospitals of treatment.

For registrations recorded as untreated, additional enquiries were made to the relevant hospitals to confirm that these lung cancer cases were in fact untreated. Also, the untreated cases who were alive at last NHS Central Registry follow-up were later individually followed-up through the cancer registration officers. For the 667 untreated cancer registrations only 5 were still alive at last follow-up.

Survival rate computations were made using the Hewlett Packard 85 desktop microprocessor of the Westminster Hospital Cancer Registry, Mould (1982), and calculated by a life-table method with data grouped into equal time intervals. The relative survival rates were calculated using the Chester Beatty Research Institute (1974) Serial Abridged Life Tables for England and Wales which are also used by OPCS (1980, 1982).

The age distribution of the 3039 registrations treated for carcinoma of the lung is remarkably similar for males and females, with mean ages respectively 64·5 years and 64·2 years. For the 667 untreated registrations the mean ages are some four years older, 68·1 years for both males and females. These mean ages compare with 66·9 years and 66·3 years for the 1977 OPCS cancer incidence data for males and females, England and Wales, but are higher than those quoted for the United States 1961–1973 USDHHS (1976) which are 62 years for males and 59 years for females. The mean ages for each sex for the three main histological groups, squamous cell carcinoma, oat cell carcinoma and adenocarcinoma, do not differ by greater than 3·5 years.

The crude survival rates for untreated carcinoma of the lung in males and females are shown in Fig. 1 for 0–12 months. The one-year relative survival rates for males and females are 9·9% and 6·1% compared with the 9·3% and 5·9% crude 1-year rates.

Fig. 2 shows the crude survival rates for 0–60 months for treated carcinoma of the lung in males and females. The 5-year relative survival rates for males and females are 10·7% and 8·3% compared with the 8·6% and 7·3% crude 5-year rates.

Table I shows the crude and relative 1-year and 5-year survival rates for 3039 treated lung cancer registrations, grouped by sex and histology. Some publications use oat carcinoma synonymously with small cell carcinoma, but since for males 238 registrations were specifically recorded as oat cell compared with only 29 as small cell, and for females 196 oat cell compared with 23 small cell, it was decided to retain the original record descriptions and analyse in terms of specified oat cell carcinoma. Other histological groups such as large cell carcinoma and alveolar cell carcinoma were too small in sample size for analysis.

The most extensive published data on untreated cancer is due to Greenwood (1926) and Shimkin (1951) but neither includes data for untreated lung cancer. Fig. 1 is therefore the only available series of published untreated lung cancer survival rates to date. The 3·4% difference in 1-year survival rates between males

TABLE.—*Crude and relative percentage survival rates of histologically proven treated lung cancer, by sex and histological group (± s.e. is given in brackets for each crude survival rate)*

| Sex | Histology | No. of registrations | 1-year survival rates (%) Crude | 1-year survival rates (%) Relative | 5-year survival rates (%) Crude | 5-year survival rates (%) Relative |
|---|---|---|---|---|---|---|
| Male | Squamous cell ca. | 1037 | 39·1 (±1·5) | 41·2 | 12·4 (±1·1) | 15·9 |
| ,, | Adenocarcinoma | 164 | 36·0 (±3·8) | 37·3 | 10·8 (±2·5) | 13·3 |
| ,, | Oat cell ca. | 238 | 13·5 (±2·2) | 14·0 | 2·3 (±1·0) | 2·9 |
| Female | Squamous cell ca. | 311 | 34·6 (±2·7) | 35·4 | 10·5 (±2·1) | 12·1 |
| ,, | Adenocarcinoma | 90 | 28·9 (±4·8) | 29·5 | 16·9 (±4·0) | 19·1 |
| ,, | Oat cell ca. | 196 | 14·0 (±2·5) | 14·3 | 3·1 (±1·3) | 3·5 |
| | | Total = 2036 | | | | |

FIG. 1.—Crude survival rates of untreated carcinoma of the lung in males and females. The relative one-year survival rates for males and females are respectively 9·9% and 6·1%. (*Note.* Crude rates are plotted in Figures 1 and 2 since these can be computed every month or every 3 months, whereas relative rates can only be computed annually since the CBRI (1974) data only provides $_np_x$ probabilities for integral numbers of years between one and five.)

and females is probably due to an excess of oat cell carcinoma registrations in the female series.

One of the recent clinical trials for treatment of small cell carcinoma was that by the Medical Research Council (1979), and it is interesting to note that they quote an 18% 1-year survival rate for patients treated by radiotherapy, of whom 66% were aged between 55 and 70 years. For oat cell carcinoma in the North West Thames region there is a 13·5% 1-year survival, Table. Of this group of 238 oat cell carcinomas in the Table, 87 were in the age group 58–67 years and the crude 1-year survival rate for this sub-group was 18·4% (±4·2%), which is in agreement with the 18% rate for the Medical

FIG. 2.—Crude survival rates of treated carcinoma of the lung in males and females. The relative one-year survival rates for males and females are respectively 30·8% and 25·1%.

Research Council trial. Livingston *et al.* (1978) quote a higher 1-year rate of 22% for 73 cases treated by radiotherapy and for a series of 479 cases reviewed by Hansen *et al.* (1980) which combined several groups of small cell carcinoma treated by various chemotherapy or combination radiotherapy regimes, a 21% 18-month survival is quoted for patients with regional disease. This compares with 9·4% and 8·4% 18-month crude survivals in females and males for the series of 238 and 196 registrations in the Table.

For a disease such as lung cancer, the 5-year survival rate is clearly not a good criterion of treatment success and a 1-year rate is more realistic. For example, for both males and females, the improvement in the 1-year survival rate is some 20% when the patients are treated, compared with when they are untreated. Differences between survivals for males and females is not large for any histological sub-group. However, for adenocarcinoma, the 1-year relative rates differ by more than 5%. In this instance, males have a 1-year survival rate 8% higher than for females, although when the 5-year survival rates are considered, survival is 6% higher for females than for males. A similar difference was also reported in the United States (USDHHS, 1976), for adenocarcinoma, 1960–1973, with a 10% 5-year relative survival rate for males and a 14% 5-year relative survival rate for females.

This review refers only to histologically confirmed registrations since these are "good" data in that a verified histology has been recorded in the routine registration system with sufficient accuracy to enable a survival analysis by histological group to be made. Also, the population of the North West Thames region is such that the regional data can be regarded as a reasonably representative sample from England and Wales. The most recently published OPCS (1982) survival statistics for England and Wales are for 1975 and give 20·0% and 17·5% 1-year relative survival rates for males and females, and 7·0% and 6·6% 5-year relative survival rates for males and females. These are lower than the rates for this North West Thames Regional *treated* lung cancer series, which have already been stated as respectively 30·8%, 25·1%, 10·7% and 8·3%. However, this is not surprising since the OPCS analysis is for a combined series of treated *and untreated* registrations, as well as for histologically confirmed *and unconfirmed* registrations.

In the North West Thames Region, a significant number of lung cancer registrations are untreated and it is probable that this pattern is repeated throughout England and Wales. Analysis of lung cancer registrations for survival patterns should therefore be made on a basis of treated and untreated series of cases, otherwise a combination of all the registrations cannot be assumed to provide the best estimate of the overall results of treatment within a large population such as a region. When data on histological verification are recorded this should be used for survival analysis, rather than be discarded, as at present occurs at OPCS for England and Wales.

A large amount of helpful co-operation was received from many people in the North West Thames Regional offices and in the individual hospitals within the region. In particular we should like to thank: Miss E. Kippen of the North West Thames Cancer Registry and Dr Y. Hollis for help and encouragement. Mr S. Ray for assistance with the abstraction of the records. Mrs P. Ratcliffe and her colleagues Mr J. Lloyd, Mr T. Anderson and Miss A. M. Spofforth, in the NHS Central Registry, for the excellent follow-up they achieved. The cancer registration and medical records officers at the various hospitals: Mrs Preston (Harefield), Mrs Dillon (Mount Vernon), Mrs Hart (Colindale), Miss O'Donnell (Charing Cross), Mrs Caines (Edgware), Mrs Tackley (Middlesex) and Miss Davies (St Mary's Hospitals). The OPCS was very helpful in allowing us to view prior to publication, the data for lung cancer in *Series MB1, no. 9.* We also thank Miss A. Jeffries for artwork and Mrs V. Chapman for secretarial assistance. Finally, we acknowledge the support and encouragement received from the North West Thames Region and from Westminster Hospital.

### REFERENCES

Chester Beatty Research Institute (1974) Serial Abridged Life Tables, England and Wales, 1841–1970, Part II, Interpolated Cohort Life Tables, Survival probability values to 1, 2, 3, 4 and 5 years from each age from 5 years to 97 years. *The Chester Beatty Research Institute, Royal Cancer Hospital, London.* (Unpublished).

Greenwood, M. (1926) A report on the natural duration of cancer. *Reports on Public Health and Medical Subjects,* **No. 33,** *Ministry of Health.* London: HMSO.

Hansen, M., Hansen, H. H. & Dombernowsky, P. (1980) Long-term survival in small cell carcinoma of the lung. *JAMA,* **244,** 246.

Livingston, R. B., Moore, T. N., Heilbrun, L. & 4 others (1978) Small-cell carcinoma of the lung: combined chemotherapy and radiotherapy. *Ann. Intern. Med.* **88,** 194.

Medical Research Council (1979) Radiotherapy alone or with chemotherapy in the treatment of small-cell carcinoma of the lung. Medical Research Council Lung Cancer Working Party. *Br. J. Cancer,* **40,** 1.

Mould, R. F. (1982) The Westminster Hospital Microprocessor Cancer Registry. *Br. J. Radiol.* **55,** 897.

OPCS (1980) Cancer statistical survival, 1971–1973 registrations, England and Wales. *Office of Population Censuses and Surveys, Series MB1,* **No. 3,** London: HMSO.

OPCS (1982) Cancer statistics survival, 1971–1975 registrations, England and Wales. *Office of Population Censuses and Surveys, Series MB1,* **No. 9,** London: HMSO.

Shimkin, M. B. (1951) Duration of life in untreated cancer. *Cancer,* **4,** 1.

USDHHS (1976) Cancer patient survival, report number 5. *End Results Section, Biometry Branch, National Cancer Institute,* Bethesda: U.S. Department of Health and Human Services, NIH Publ. No. 81–992.


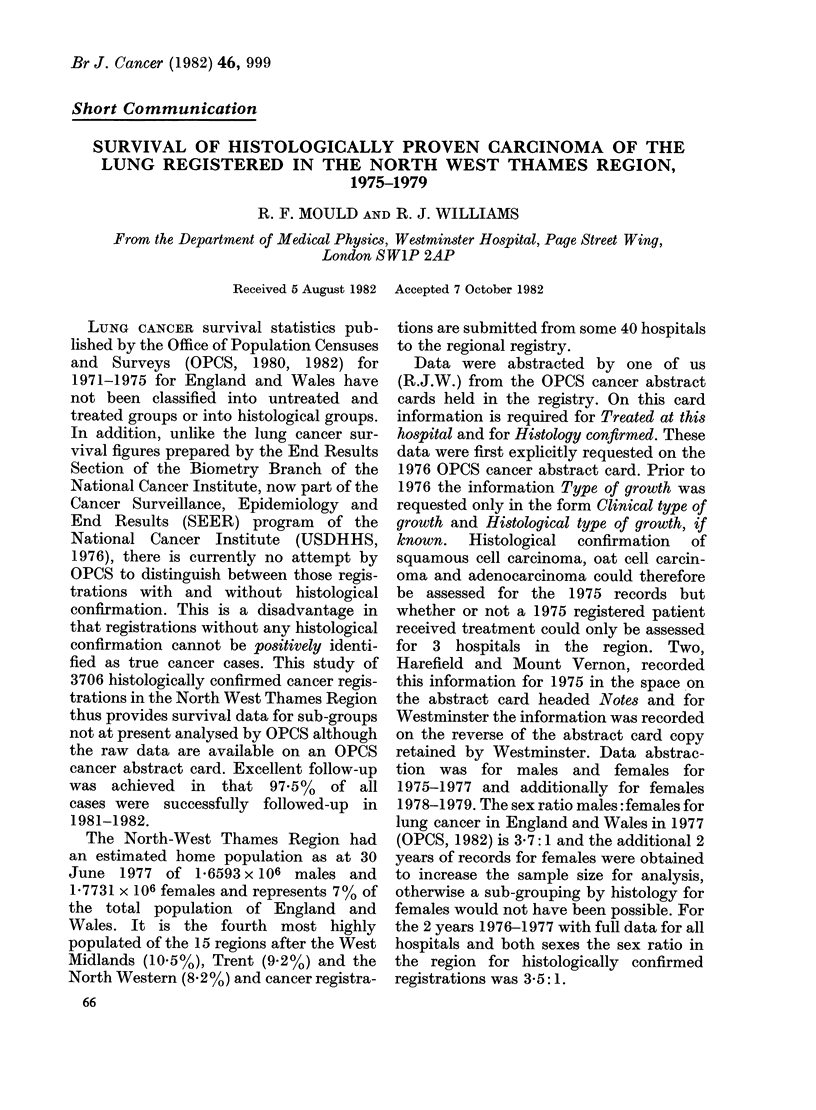

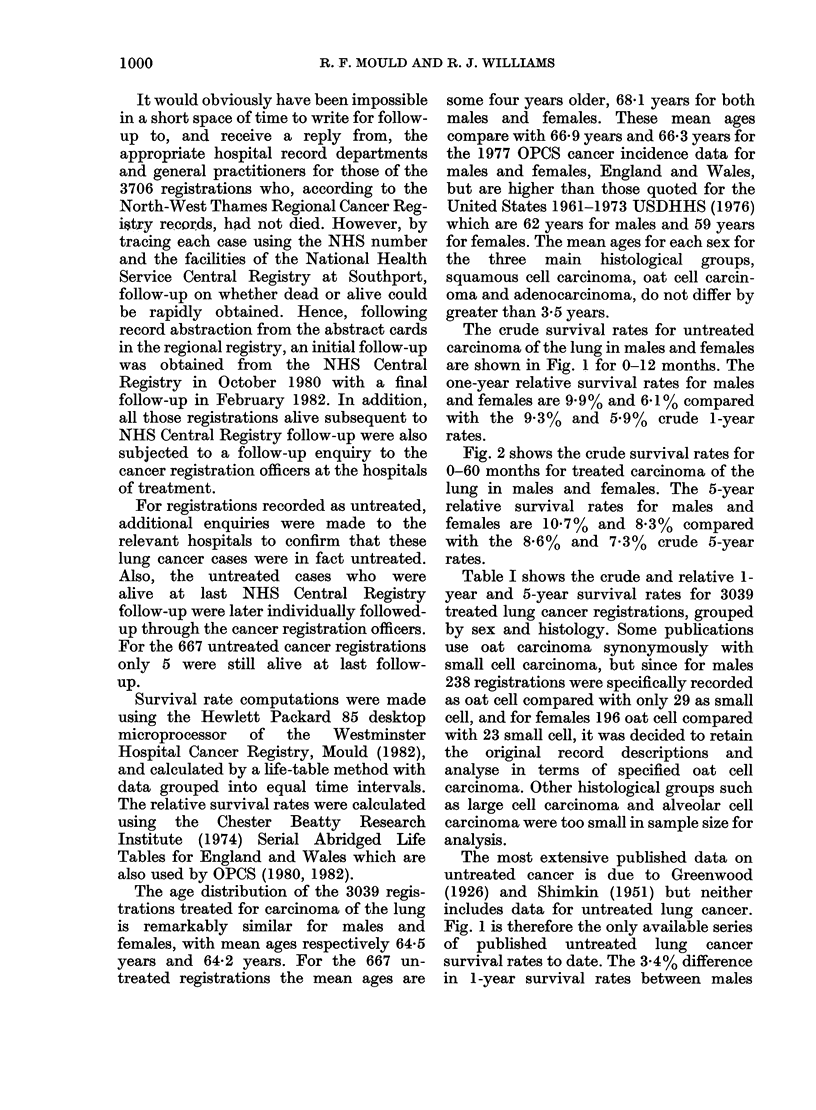

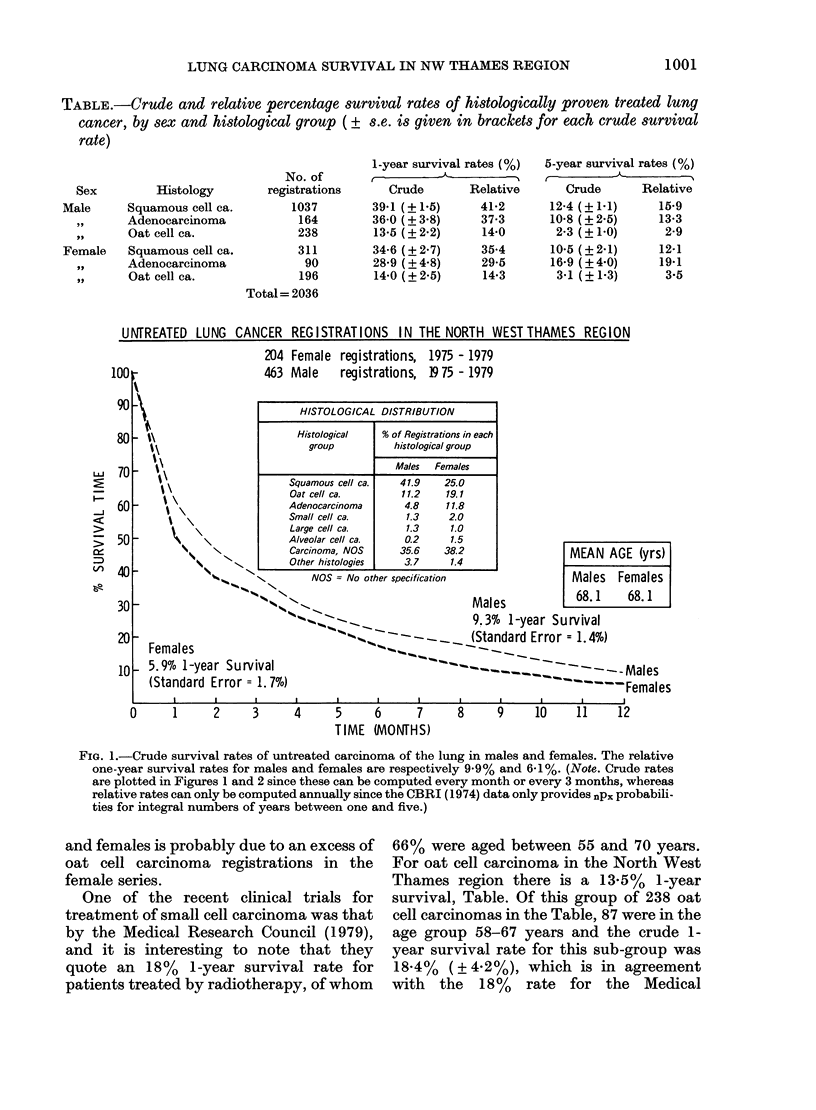

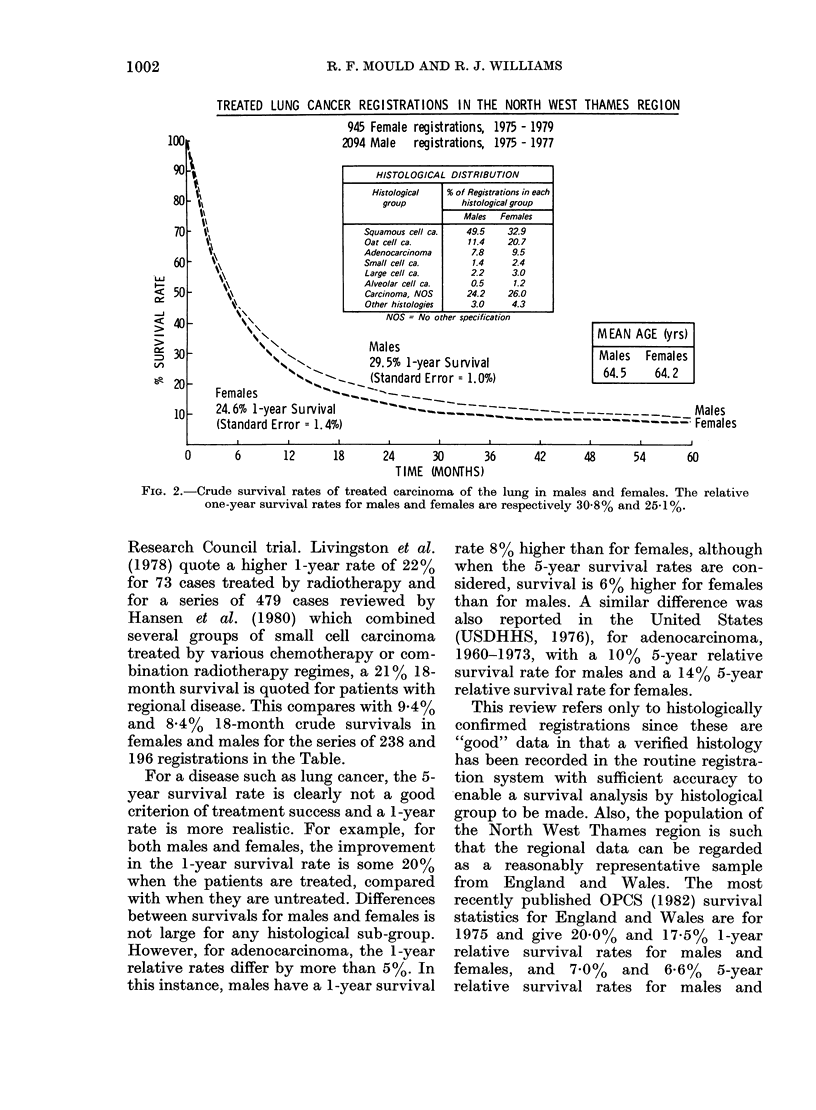

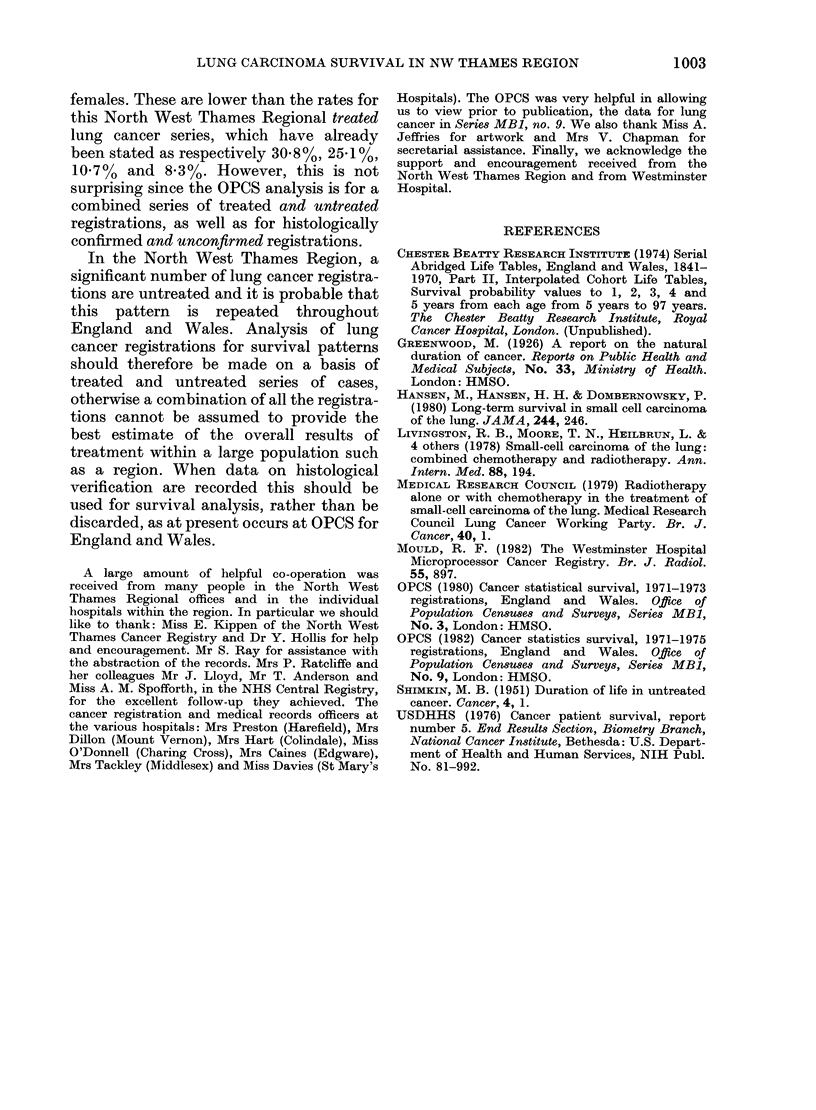


## References

[OCR_00594] Livingston R. B., Moore T. N., Heilbrun L., Bottomley R., Lehane D., Rivkin S. E., Thigpen T. (1978). Small-cell carcinoma of the lung: combined chemotherapy and radiation: a Southwest Oncology Group study.. Ann Intern Med.

[OCR_00607] Mould R. F. (1982). The Westminster hospital microprocessor cancer registry.. Br J Radiol.

[OCR_00624] SHIMKIN M. B. (1951). Duration of life in untreated cancer.. Cancer.

